# End-of-life decisions: A focus group study with German health professionals from human and veterinary medicine

**DOI:** 10.3389/fvets.2023.1044561

**Published:** 2023-02-15

**Authors:** Felicitas Selter, Kirsten Persson, Peter Kunzmann, Gerald Neitzke

**Affiliations:** ^1^Institute for Ethics, History and Philosophy of Medicine, Hannover Medical School, Hannover, Germany; ^2^Applied Ethics in Veterinary Medicine Group, Institute for Animal Hygiene, Animal Welfare and Farm Animal Behavior, University of Veterinary Medicine Hannover Foundation, Hannover, Germany

**Keywords:** veterinary ethics, medical ethics, death and dying, euthanasia, physician-assisted death, interdisciplinary focus groups, qualitative research, heterogeneous focus groups

## Abstract

**Introduction:**

At first glance, human and (companion animal) veterinary medicine share challenging processes in end-of-life (EOL) decision-making. At the same time, treatment options in both professions are substantially different. The potential of an interdisciplinary exchange between both fields has been neglected by empirical research so far.

**Methods:**

In this qualitative study, professionals from both fields were brought together in interdisciplinary focus groups to investigate the ethical aspects of convergences and divergences in EOL situations in human and veterinary medicine. The authors present and discuss an innovative mix of materials and methods as stimuli for discussion and for generating hypotheses.

**Results:**

The results point toward a general convergence of issues, challenges, and judgements in EOL situations in both fields, such as professional ethos, communication with the family and the role thereof as well as the ideals of death, clearly exceeding the expectations of study participants. At the same time, the study highlights a few prominent differences such as the access to patients' preferences or legal and practical constraints.

**Discussion:**

The findings suggest that using social science methods in empirical interdisciplinary biomedical-veterinary ethics could help to shed more light on this new area. Animal as well as human patients can potentially benefit from this mutual, scientifically accompanied exchange and the resulting identification and corrections of misconceptions.

## 1. Introduction

Both palliative care providers in human medicine and veterinarians in small veterinary practice are confronted with EOL decision-making on a regular basis. On the one hand, human medicine and small veterinary practice considerably differ from one another with regard to, for example, financial constraints and infrastructure. On the other hand, small animals such as dogs and cats have a much higher life expectancy nowadays (including the age-related problems associated with an extended lifespan) than before due to better diet, veterinary medical progress, veterinary medicine specialization, urban animal management, and pets' societal status as family members (1, 2). This has led to a convergence of EOL situations in small animal medicine with human medicine. As a consequence, ethical issues surrounding EOL decision-making in small animal practices and human medical hospitals share important similarities regarding, for example, quality of life (3–6), life-prolonging therapies (7, 8) and moral distress (2, 9, 10). While prevailing conditions can be quite similar, outcomes are conceivably different. Human euthanasia is illegal in Germany (as in most countries worldwide), even though a liberal approach to physician-assisted suicide has been underpinned by recent court decisions (11). In stark contrast, pets are typically euthanized when their estimated quality of life deteriorates (even though pet owners and veterinarians might disagree about the assessment and decision). German animal protection law demands a justifying reason (“*vernünftiger Grund*,” §1 German Animal Welfare Act) for killing animals. Therefore, veterinarians are legally obliged to judge the animal's suffering as unbearable before euthanizing her or him. Regarding interdisciplinary references, an asymmetry can be identified in the literature. Veterinarians, on the one hand, occasionally comment (critically) on EOL situations in human medicine (2, 12). However, the discussion is mostly *about* human physicians, but less *with* them. In human medicine, on the other hand, hardly any human physician comments on EOL decisions made in small animal veterinary practices, and, if so, rather as an affected animal owner (13).

For this reason, we brought together human and veterinary healthcare providers in a standardized setting of a focus group study and had them discuss what they consider a good death for their own patients but also for the patients of the other profession. Ideals of dying, criteria of EOL decision-making as well as experiences and knowledge from both professions should meet.

Fracture lines were expected to be found both between and within disciplines, for example, between clinic and practice, between different hierarchical levels, or between palliative and curative approaches of both medical fields. At the same time, a dialogue and an outside perspective might initiate a critical reflection on terms, concepts, and procedures in one's own profession among both veterinary and human medical personnel.

In this article, the focus will be on our methodological approach, which we consider exceptional in veterinary social science regarding, first, the heterogeneous compilation of our focus groups and, second, the mix of discussion inputs (from here on “materials”; see below for details). Together with a relatively strict moderating technique in comparison to other focus group studies, this methodological approach allowed us to gain responses from the participants on their perspectives about concepts and practices in the respective other profession both before and after group discussions on the subject.

Hence, we present and discuss two research foci. Firstly a methodological aim, namely the introduction of an interdisciplinary approach in veterinary social science and, secondly, the discussion of a health-care professionals' perspective on EOL situations in human and veterinary medicine. This study is methodologically exceptional insofar that the authors used a rather uncommon approach by interviewing professionals from human and veterinary medicine together in heterogeneous focus groups and by directly asking participants for their views and experiences regarding EOL situations in their own but also in the respective “other” profession.

## 2. Methods

The focus group study was embedded in the larger interdisciplinary project “Dying like a dog,” looking at convergences and divergences in human and veterinary medicine. After reviewing literature and conceptually analyzing EOL situations and potential transfers between both fields (14–17), the above-mentioned research questions emerged that called for empirical investigation. How do the stakeholders discuss convergences and divergences when they are confronted with situations and professionals of the respective other discipline? What is their attitude toward the legal and informal standards, the relations between patients and healthcare providers, potential transfer of concepts, and the opportunity to learn from exchange with the other professional field?

Focus group studies usually assemble stakeholders of one field who often have a similar professional background or common experience. For our research questions, as explained above, we decided to bring together professionals from two different fields (veterinary and human medicine) and different professional roles, i.e., (veterinary) nurses, physicians/veterinarians and further healthcare providers. This created, on the one hand, the opportunity to debunk mutual prejudices regarding EOL routines in each field, to clarify misconceptions regarding terminology and practice, and to exchange assumptions regarding constraints and motivations in the respective other field. On the other hand, the study gave room to find out about commonalities in both professions, for example, regarding emotions, relationships and interaction between different stakeholders in the process, communication and justifications for decisions and actions. Our main interest were the following questions: Which similarities and differences are perceived by our stakeholders to which extent? How are these explained and justified? The focus group study consisted entirely of heterogeneously composed groups, because we expected convergent and divergent perspectives to better emerge in joint discussions.

The decision to have physicians and veterinarians in one group, nurses and veterinary nurses in another allowed us to shift attention away from the joint expertise of treating patients–as would be typical in a focus group study–to the characteristics that make EOL situations and accompanying ethical deliberations in the two professional fields comparable or different. We thus intended to explore challenges and benefits by providing insight into and analysis of different passages of our uncommon focus group discussions. Expected outcomes were:

- Potential misunderstandings regarding terms and concepts in the other discipline that–in the in-depth discussion–turn out to differ from the participants' assumptions and expectations. These might, among other things, be based on explicit or implicit prejudices against the other discipline;
- A joint discussion of exemplary situations in both veterinary and human medical practice including general judgements on the moral status of humans and non-human animals or, more precisely, on important decision criteria when treating (non)human patients;
- A refined perspective on the other discipline after the focus group.

Several materials were implemented in the study, which,

- Created a shared foundation for the participants with different professional backgrounds to discuss interactively;
- Pointed at crucial questions and issues related to the research questions of the overarching project, i.e., convergences and divergences in EOL situations in human and veterinary practice, with an intended richness of associated topics and depth of prompted discussions.

### 2.1. Recruitment and setting

Participants were recruited from all over Germany *via* professional websites of clinics and practices, and *via* the professional networks of the project team members using mailing lists and individual email contacts. Potential participants were identified by screening for professional specializations that suggest that a person works with patients in end-of-life situations. For human medicine that included general practitioners, palliative physicians and nurses, practitioners and nurses working at geriatric units, in oncology or in ICUs. For veterinary medicine, we included veterinary nurses and practitioners in clinics and practices. Seven focus groups convened between July and November 2020. With the exception of the first focus group, the discussions took place as online meetings *via* Microsoft Teams due to pandemic restrictions. All participants signed an informed consent document (see Appendix). All meetings took ~2 h, were recorded, and the audio files were transcribed verbatim by a transcription service provider. Video files were not used for the analysis and were deleted after extracting the audio files. Transcripts used pseudonyms and were coded and analyzed by two members of the team (FS, KP).

### 2.2. Group composition

Each group consisted of three to five participants (26 in total) and was moderated by one member of the team (KP, FS alternately) and co-moderated by the other. Three groups consisted of nurses and veterinary nurses [i.e., “*Tiermedizinische Fachangestellte*” (German job title)], four groups of physicians, veterinarians, an alternative animal health practitioner, and a psychologist. For an overview of group compositions, see Table 1.

**Table 1:** Composition of the focus groups, pseudonyms for participants “HX.Y” or “VX.Y” (“H” for human medical staff, “V” for veterinary staff; first number X corresponds to the focus group number, second number Y to the counting list starting with 1).

**Focus group**	**Human medical personnel**	**Veterinary medical personnel**	**Number of participants**
**1**	H1.1, H2.2 physicians	V1.1, V1.2 veterinarians	4
**2**	H2.1 physician	V2.1, V2.2 veterinarians	3
**3**	H3.1, H3.2 nurses	V3.1, V3.2 veterinary nurses	4
**4**	H4.1, H4.2 nurses	V4.1, V4.2 veterinary nurses	4
**5**	H5.1, H5.2 nurses	V5.1, V5.2, V5.3 veterinary nurses	5
**6**	H6.1 psychologist	V6.1, V6.2 veterinarians	3
**7**	H7.1 physician	V7.1 Veterinarian V7.2 Alternative animal health practitioner	3

### 2.3. Interview guide

The interview guide consisted of four major chapters with additional sub-parts (see Appendix). The first two major chapters each presented a conversation starter, providing controversial examples from each discipline. The first example introduced a veterinarian and her two clients (mother and adult daughter) in a 5-min video clip from a German reality TV-show (18). The scene depicted the vet delivering a terminal diagnosis regarding an elderly dog to her owners in the waiting area of the veterinary practice. The interaction of the vet with the two overwhelmed owners was meant to provide the basis for an exchange about the appropriate setting for delivering diagnoses, the nature of communication in EOL situations, the involvement of clients in therapy decisions, decision-making criteria for elderly patients etc. The video clip was presented to create a shared experience among the participants and to prompt discussion. By starting with the reactions of human medical personnel to the scene, we captured reactions from people outside of the focused field first without already biasing these reactions through the responses of the professionals. This procedure was repeated in the second chapter, only with swapped roles. Here, we introduced participants to a newspaper article about a case of killing on request in the Netherlands (19). A 74-year-old woman had ruled in advance (written living will) that she wanted to be euthanized in case of progression of her dementia. Whenever asked, she said she did not want to die yet. Her doctor and family, however, determined one day that the state she had described in her living will had been reached. Therefore, the woman was euthanized, while still verbally complaining and fighting back whilst being administered with the injecting initiating death.

For the third part of the interview guide, three PowerPoint slides were prepared, each introducing a fictitious person uttering a statement regarding (1) terminal care for companion animals, (2) euthanasia of animals, and (3) high-tech veterinary medicine. A translated version of the slides is provided in the Appendix. The slides covered all topics the project team believed to be crucial for debating EOL situations. The fictitious persons on the slides served as outsiders giving their sometimes provocative, polarizing or minority opinion. In contrast to direct questions by the moderator, this method gave the opportunity to strongly (dis)agree with or criticize a position without having to take into account the other person's feelings and judgement. The slides were only used in case the topic had not been addressed earlier in the discussion and there was enough time left.

In the final chapter of the interview guide, participants were instructed to work with Padlet (20). Participants were asked as a group to think of the ideal human or companion animal death and assign some key words written on virtual notes to either “human death” or to “animal death” (or both) and discuss their decisions. Participants also had the option to create additional virtual notes with their own key words. A complete list of the provided key words can be found in the Appendix. Our aim was to clarify whether participants perceived the characteristics/conditions of good dying as more or less similar for humans and companion animals in light of their previous discussion.

### 2.4. Analysis

The analysis was targeted at both the method of using multidisciplinary focus groups and the thematic content regarding EOL situations. Therefore, all transcripts were repeatedly surveyed for both aspects of discussion dynamics and thematic patterns. Two authors (KP, FS) conducted the analysis. The authors have a background in animal ethics and philosophy (FS) and empirical bioethics and philosophy (KP) and consulted a social scientist to critically look at their interview guide and analytic focus (see acknowledgments). The team was complemented by a student intern with a background in philosophy and media science who provided an additional perspective on the data. Based on more recent work of Braun and Clarke, the authors' methodology could be called “codebook thematic analysis” (21). The steps of the content analysis were carried out as follows. The authors independently read the transcripts several times and took notes for each group regarding,

- Reiterating themes
- Reaction to discussion inputs (for example, Did the TV clip start a controversial discussion? How did the group react to the statements? Did they agree on key words in the Padlet task as a group or did they rather work on the task individually?)
- Interdisciplinary dialogue (for example, Did participants comment on/question/agree with processes, habits or attitudes that were presented in the material? How did they react to the other discipline's participants' statements? Did they directly or indirectly compare both disciplines based on their own professional experience?)

After the first read-through, the authors exchanged notes and thoughts before starting the interpretative coding. While generating themes during the analysis, the authors' focus lay on participants' interaction due to the interdisciplinary group composition and on topics generated from previous work of the authors in the same project (14, 15, 22). In a first step, both authors conducted interpretative coding independently for a sample of two focus group transcripts. The identified codes were then compared and adapted, building a common coding scheme that was still flexible regarding modifications. Regular meetings and discussions between the coding authors revealed (mostly) similar and (on few occasions) different interpretations of statements, reactions, and non-verbalized attitudes and led to a broad spectrum of themes. Exemplary passages for the themes were identified in mutual discussion. Both inductive and deductive coding was used. As indicated in the research questions, the effect of bringing both professions together was expected to address topics beyond the analytical research results the authors had worked on before (inductive approach). In addition, themes from the above-mentioned previous work provided a basis for deductive analysis.

Participants' verbal quotations in this article were initially translated with DeepL (https://www.deepl.com/translator) and linguistically and grammatically adjusted if necessary by the co-authors and a native English speaker (fluent in German). The position indicators for quotations refer to the transcripts.

## 3. Results

Pseudonyms were attributed to each participant, using the focus group number and their professional field (cf. Table 1). The results will be presented in line with the above-mentioned research questions, i.e., first general themes and patterns (3.1) and then reflections on both professions and their patients (3.2).

### 3.1. General themes and patterns

#### 3.1.1. Communication in veterinary medicine

By first asking healthcare professionals from human medicine for their impressions on this case, we intended to capture non-biased (i.e., here: unaffected by immediately preceding expert opinions) from people outside veterinary medicine. This procedure proved to be effective, as analysis revealed a robust pattern across all seven focus groups.

Theme: Approval and disapproval of communication

Some human healthcare professionals described the vet quite positively as “*authentic”* and “*empathic” (H1.2, pos. 44*) and the scene as “*appealing” (H1.2, pos. 44)* and “*familial” (H7.1, pos. 48)*. H5.2 appreciated that she “*did not beat around the bush, but formulated everything very directly” (pos. 66)*. Others expressed restraint or mild criticism on how the diagnosis was delivered to the two overwhelmed dog owners and found it “*a little astonishing the way she brought it across with the diagnosis” (H4.2, pos. 52)*, “*quite, quite rational and quite direct” (H3.2, pos. 61)* as well as “*concise”* and “*relatively casual” (H2.1, pos. 65)*. H3.2 stressed, however, that the vet “*became a bit more warm-hearted”* during the scene and “*also briefly put her arm around the dog's owner” (pos. 61)*.

In stark contrast, participants with a background in veterinary medicine strongly disapproved of the TV vet's handling of the situation in the depicted scene, stating they “*would never do it that way” (V6.1, pos. 52)* as “*[s]he actually beat them to death with what she said” (V7.1, pos. 52)*.

Participants also negatively noted that the veterinarian failed to lay out the possible treatment options, including operation, and did not support the owners in making a decision but instead made the decision for them *(V6.2, pos. 53)*.

Two human medical professionals, however, also criticized the TV vet comparatively harshly as “*very nonchalant* [German: ‘burschikos'*]” (H1.1, pos. 43)* and “*terrible” (H6.1, pos. 50)* in her communicative approach. Both referred to their own professional expertise in human medicine and transferred it to veterinary medicine: “*I would in humans, or / Yes, even if I were a veterinarian, I think I would convey the diagnosis differently than just in the waiting room after a brief pat down” (H1.1, pos. 43)*. In a similar fashion, H6.1 stated that “*as a palliative care provider* [German: “Palliativgewächs”]*, one is naturally used to a very gentle approach and is also very well-trained in this. And it was a disaster what I saw there. And in my opinion, this is also not possible in veterinary medicine. […] You can't deal with it like that. Really. Yes. An absolute crisis.” (pos. 50)*.

After listening to the harsh criticism of the two veterinarians in her group, H2.1 revealed that “*I didn't even dare to say at the beginning that I found it* [the setting] *so unusual” (pos. 76)*.

Theme: Communicative differences in human and veterinary medicine

Some human healthcare providers with a well-meaning attitude toward the TV vet considered corresponding EOL situations and the associated requirements for healthcare providers in both disciplines rather differently, arguing that “*[h]uman medicine [is] different, that only works for veterinary medicine, but there it is good” (H1.2, pos. 44)*. H3.2 was “*a bit shocked at first”* but immediately qualified this initial reaction: “*But of course I can imagine that in the veterinary field it is not quite as sensitive a topic as when you have to deliver a terminal cancer diagnosis to a human being” (H3.2, pos. 61)*. Another nurse felt that “*[i]t would be good if it were sometimes so direct*” in human healthcare, seeing as “*our doctors are then rather beating around the bush” (H5.2, pos. 68)*.

#### 3.1.2. Euthanasia of a woman with dementia

Analogous to the proceeding in the first case example, participants from outside the relevant profession (in this case, human medicine) were asked for their impressions. The method was effective insofar that lively discussions were stimulated in most groups. In contrast to the video clip example, no pattern along the fracture lines between human and veterinary medicine became apparent. Instead, two patterns were conceptualized that could be attributed to the professional groups within a discipline, i.e., (mostly) doctors from human and veterinary medicine on the one hand and (veterinary) nurses and caregivers on the other hand. Focus groups 1, 2, 6, and 7 (pattern A) strongly disapproved of the Dutch physician's course of action; groups 3, 4, and 5 (pattern B) were highly ambivalent.

Theme: Disapproval of physician's actions

Within pattern A, participants especially took offense at the execution, i.e., the administration of a respiratory paralytic agent, while the patient was still conscious. They described the procedure as “*worst case” (H2.1, pos. 93)*, “*rather outrageous”* and “*really horrible” (H7.1, pos. 76), “something that totally moves us, where we can't go along with it at all” (H6.1, pos. 89)* and expressed shock that “*[t]he poor woman suffocated” (V6.2, pos. 83)*. It was also perceived as “*murder” (V1.2., pos. 84)*.

Human medical professionals emphasized that even under the assumption that the euthanasia of dementia patients can sometimes be justifiable, there were no signs for suffering in the described patient at the moment of her euthanasia; “*[o]n the contrary, she rather had something life-affirming in this situation” (H7.1, pos. 76)*. H6.1 wondered whether in spite of the existence of a living will, “*is it not possible to grant her a certain degree of decision-making capacity, even though she has dementia?” (pos. 89)*

Some participants were certain that the course of action did not represent the patient's wish *(H6.1, pos. 89)* and speculated that “*this is euthanasia for the relatives, not for the patient” (H1.2, pos. 88) “because that's the better option for them, or because they somehow can't bear it anymore, the condition of the woman. Or because it's exhausting for them” (V6.1, pos. 82)*.

Theme: Ambivalence toward physician's actions

In the groups consisting of (veterinary) nurses and other caregivers, several participants explicitly conceded their ambivalence (for example, *H3.2, pos. 134; V4.1, pos. 106; H5.1, pos. 103*). In other cases, analysis revealed ambivalent or inconsistent stances. As an especially striking example, veterinary nurse V4.2 initially expressed that she was “*able to relate to why the doctor acted the way she did” (pos. 105)*, but then revised her first impression as a consequence of another participant's contribution. Now agreeing that the current wish should have priority over the preconceived wish of the patient, she expressed her uncertainty regarding a correct interpretation of the patient's aversive behavior:

“*The question is: Why is she resisting? Is she perhaps generally afraid of injections, of infusions? […] Why might she be upset? Was it because of the execution of euthanasia or just because a doctor comes with an injection? But it's difficult. When someone is so opposed to it. I don't know if I could have forgiven the doctor in the case of my grandmother” (pos. 110)*.

This ambivalence was also apparent in other focus groups, occurring between two participants. A veterinarian stated:

“*So my first thought was, ‘Oh my God, I think it's quite creepy, that idea.' And the second thought was: ‘Actually we don't do anything else with every euthanasia of animals, if you like'. Because every animal is fixated somehow […]. Nobody knows […] if it wants to die, or if it wants to live on, with appropriate medication. But somehow […] in my head there is a difference” (V1.1, pos. 81)*.

Another veterinarian in the same group disagreed, strongly arguing that “*[i]f an animal is still able to resist so much and is so upset, then it is not my job to kill the animal now, because then there is no justifying reason* [German: “vernünftiger Grund”] *and then I would commit a crime. Whether with the human being or with the animal” (V1.2, pos. 84)*.

Theme: Approval of physician's actions

As was the case in the first example, analysis revealed interesting deviations from the patterns. Caregivers H3.1 and H4.2 were remarkably positive about the doctor's approach, placing emphasis on the (autonomous) patient's right to self-determination. This can also be explained by their reported professional experiences–both described dying processes, which, from their point of view, were negative. H4.2 reported that doctors had disregarded living wills or the presumed will of the patient *(pos. 108)*. H3.1 was frequently confronted with the mental deterioration of nursing home residents, which he perceived very negatively.

#### 3.1.3. Animal hospice and palliative care

To enable comparison across groups, one statement was singled out for analysis that was used in each group, i.e., terminal care and hospice for companion animals. The PowerPoint slide presented a drawing of a woman and a dog on a leash. The woman says, “*When the time has come for Waldi, I would like terminal palliative care for him. I have already discussed this with my veterinarian. Just killing him when he can't really go on anymore*–*that's not for me! We let elderly people go in dignity as well and human medicine makes dignified dying without pain and fear possible. So, that should be a given for our companion animals, too!*” [translated from German].

The term *terminal care* (German: “*Sterbebegleitung*”) in the statement was intended to make participants aware of a possible transfer of EOL care options (including hospice) from human to veterinary medicine but simultaneously not rule out the option of euthanasia completely. The reason for this was that we were particularly interested in how the participants would interpret the woman's request for terminal palliative care, for example, as a wish for palliative care and then euthanasia, or for a “natural” death. All groups ultimately agreed on the term referring to a *hospice-supported natural death*, i.e., “the treatment of pain and other signs of discomfort under veterinary supervision until the natural death of the individual” (23).

Theme: Criticism of alternatives to euthanasia in companion animals

All participants assessed the shown woman's wish for terminal palliative care for her dog predominantly negatively. However, those with a background in veterinary medicine did so significantly more negatively than participants from human medicine. V2.1 reported that “*there are more and more who are of the opinion that you would play God in such a case”* and for whom “*[e]uthanasia is not an option,”* wanting their animal “*to live until it is no longer possible” (pos. 115)*. She perceives this transformation in veterinary medicine as “*terrible in some cases” (ibid.)*.

Participants from human medicine were more ambivalent, arguing that in some situations veterinary palliative care was “*a good idea,”* while in others “*the somewhat faster variant would be more humane.”* The same person also deemed it difficult to decide, in general, “*[w]hether palliative care is now necessarily appropriate for an animal without it being able to decide whether it wants to live longer, perhaps with less pain, but perhaps still with pain and with restrictions” (H5.2, pos. 127)*.

H4.1 and H4.2, both working in intensive care units, were much more critical with regard to palliative care and hospice even for human patients, emphasizing that “*especially in hospital, there are also very inhumane manifestations of EOL care, and it's not always just sunshine and roses and we put up some candles and let some music play” (H4.1, pos. 137)*. H4.2 added that, unfortunately, in the normal day-to-day life on the ward, “*it happens far too often, that the dying person gets the short end of the stick.” (pos. 138)*. In the introductory round, nurse H4.2 explicitly blamed the “*in part inhumane conditions” (pos. 13)* at intensive care units and the fact that in veterinary EOL care, one has other options (i.e., euthanasia) for her change of profession from human to veterinary medicine.

Theme: Companion animals' hospice-supported natural death

There were deviations from the dominant theme, some of which were expected in light of the participants' backgrounds. V7.1 and V7.2, working predominantly with elderly companion animals, expressed a positive attitude toward the possibility to abstain from euthanizing the animal at the end of their life. Cross-group analysis revealed conflicting positions between participants of different groups. When referring to “*no longer dignified death[s]”*, V2.1 stated that “*especially cats […] don't die just like that, it takes forever” (pos. 115)*. In contrast, V7.2, who let her cat die rather than have her euthanized, reported that “*she just got a little less and less and really fell asleep peacefully”* and died a “*dignified death” (pos. 95)*.

#### 3.1.4. A good death for humans and animals

Participants were asked to assign key words when describing the ideal deaths of humans and companion animals and discuss their decisions when doing so. Moral intuitions on a good death differed among the participants.

Theme: Merging of perspectives and needs of the patient, the patient's family/owner and the professional staff

H4.1: *I have experienced it in the work context that the people who in the end […] ‘have found their peace with it' […] could let go better. […] I found it more pleasant and the relatives could deal with it better if they had the feeling that their dying relative was okay with it*. (pos. 188)

V5.3: *Personally, I would have left it [the key word “at home”] even more to the right side [of the Padlet, i.e., belonging clearly to the good human death], because I once had to put a dog down at my home and every time I walked past that place I thought: ‘There he was, there he was, there he was, oh God, there he was'. […] I would never do that again*. (pos. 160)

Theme: Overlap of good animal and good human death/dying

All groups agreed that many characteristics of good dying applied to humans as well as animals (for example, “*painless,” “without fear”*) but they also placed key words exclusively at “good human death” (for example, “*self-determined”*) or “good animal death” (for example, “*while sleeping”*). Even though the ideals of death of human and companion animal patients converged, significant differences remained. V3.1, in stark contrast, proposed putting all key words in the middle because “*there is nothing that I would wish for a human being that I would not wish for an animal and the other way around as well” (pos. 203)*.

There was broad consensus across all groups that good dying was a matter of individuality for humans as well as companion animals. H1.1 critically remarked that

“*[w]e have an ideal idea of how a good death should be. That we all sit around the patient and a candle burns. As a therapist, I think you always have to take a step back and really look: What does the patient want? […] [Experience teaches] to look at both humans and animals very individually: What defines the living being and what would have been the personal, individual wishes?” (pos. 223)*

Participants agreed that acceptance of death was irrelevant in case of animals but there was no consensus on whether acceptance of death is necessary for a human death to be “good.” For the nurses among the groups, patients who never accept the fact that they are dying, even though “*we as nursing staff always have to think to ourselves, ‘My God, he's got to get it sometime”' (H5.1, pos. 197)* do not die “well.” Oncologist H2.1 agreed that ongoing repression and denial on behalf of a patient “*makes it very, very difficult for others”* but disagreed that death always has to be accepted, “*because if I'm young and have small children or I'm old and still want to live, then maybe I just don't accept that I have to die. And that's maybe kind of okay too” (pos. 174)*. For H4.1, a good death entailed a confrontation with one's own mortality and the possibility of life after death. She considered this “*very important for the process of being human” (pos. 176)*. V6.2, in contrast, described an unexpected sudden death as ideal for both animals and humans *(pos. 168, 170)*–even though she partly revised this later by stating that a self-determined death is actually “*perfect” (pos. 191)* for humans.

### 3.2. Reflections on both professions and their patients

#### 3.2.1. Perspectives on end-of-life situations

Theme: Different options regarding EOL human and veterinary medicine

Comparing options in EOL situations in both disciplines, some participants from the human medical field rather clearly stated that they–or their patients–envied veterinarians for being able to “*work like that and that you can say ‘Okay, we'll end it here' in the final stage” (H3.1, pos. 63)*, i.e., for being legally allowed to euthanize their patients. Implicitly arguing for a convergence between the two fields, H5.2 states that “*unfortunately, working in an ICU sometimes entails that it seems to be more pleasant for animals to die than for humans. As sad as that sounds. That they can get the more pleasant death” (pos. 228)* and H3.1 adds that “*we are in Germany […] pretty far behind, that we do not have this possibility with euthanasia. I think that is something important for diseases like dementia” (pos. 63, 133)*.

At the same time, H3.1 was able to sympathize with patient owners' desires to administer palliative care to companion animals, because “*EOL care, as it is possible here, is definitely pretty good and the possibilities are definitely fairly good. Accordingly, I find the desire to give their animal*–*I can understand the desire” (pos. 171)*.

Those statements are not necessarily contradictory. Apparently, the participant would prefer the larger set of EOL treatment options for both human and animal patients. He voted for a convergent development, with both disciplines implementing beneficial elements of the respective other practice.

Theme: Cross-disciplinary references to EOL situations in human and veterinary practice

V7.1 reported that “*this situation*, [i.e., that people would also like to be euthanized,] *has unfortunately also happened”* to her several times, when she made house calls and “*ends up where the grandma is and maybe the grandma is already lying in the nursing bed. And then you just put the dog down, and the grandma says: ‘That's great, can't we do that for me, too?' And then, of course, you stand there, looking a bit silly” (pos. 89)*. Despite their different professions, V7.1 shared the experience of geriatric nurse H3.1 who was occasionally confronted with a human patient's wish to end their life, without being able or allowed to act or intervene.

When discussing the Dutch human euthanasia case, veterinarian V6.2 went even further, believing “*that human doctors could sometimes learn something from veterinarians. Just how do you put a human to sleep properly? […] Well, I mean, you don't put them to sleep, don't get me wrong, but how can you inject a respiratory paralyzing drug when the person is not actually under anesthesia? […] I could get upset about it now […], I don't want one of my patients to die like that” (pos. 85)*.

Theme: Reasons and justifications for differences in veterinary and human medicine

For one veterinarian, difficulties broadening their argumentative perspective beyond the contrasting legal situations in human and veterinary medicine surfaced during an intriguing passage in focus group 7. We therefore report the full dialogue:

V7.1: *Yes, that's a good question now, why no palliative sedation [in veterinary practice]. […] [S]o if I sedate the animal, then it sleeps, I would now claim. And I would then have to monitor it until it finally falls asleep. That is now for me actually also such a term clarification. I don't know exactly how this works in human medicine. I would now like to ask H7.1 another question: “Are there people who wake up again from palliative sedation?”* (pos. 105)

H7.1: *Well, […] the palliative sedation, if you use this standard term now, so of course already has the goal, […] that then actually someone no longer awakens, predominantly. Of course, we try to go as deep as necessary and as superficial as possible. […] I've already had two or three patients where the treatment was so superficial that we were able to reawaken them, and they were able to make contact with their relatives. So that's always a balancing act. We actually do monitoring […]. And that is certainly hardly possible at home in this form. I have never done this at home, it requires a lot of personnel. And that is certainly also in the veterinary medicine what would bring everything, also with safety, also very much to its limits*. (pos. 106)

The physician here believed the difference to be on a logistic level. Obviously, veterinary medicine cannot provide the same supervision and monitoring as human medicine and cannot fall back on the same amount of personnel. Several participants brought up this difference in the degree of institutionalization as a side note. However, the veterinarian implicitly returned the initial question posed by the moderator (“Why is there no palliative sedation in veterinary medicine?”), to the human physician (“Why is there no euthanasia in human medicine?”). She seemed to be convinced that the responsibility for this deficit lay with human medicine in this matter:

V7.1*: Yes, above all, I believe that human physicians are not allowed to euthanize humans, so they have to use palliative sedation. […] I would see that now simply as the difference, because the animals may be euthanized […]. And then the practically thinking veterinarian would say, “why should I sedate the dog now for 2 days or 8 h, rather, I really let him go to sleep [i.e., euthanize him] now properly.”* (pos. 107)

The physician offered an explanation here. Human medicine uses palliative sedation as the ultimate means to relieve symptoms. He confirmed that it is reversible–making it substantially different from euthanasia–but emphasizing that “*it's basically an ethical issue that's behind it,”* he insisted that the meaningful difference is in the attitude of “*the goal, not to cause the person to die” (pos. 108)*. Still, the veterinarian was not convinced. As if having fully embodied the above-mentioned legitimacy, she then suspected the physician's intentions to be shrouded by the legal prohibition:

V7.1: *Yes, in humans it is then accepted that they will die as a side effect, that is also clear. However, I really think that for legal reasons, no human physician is allowed to do it, that is, he must not want to do it. Or he must not have it as a goal and I as a veterinarian, I already have it then as a goal. So, I must also admit that there are cases where one says, it is good that I can euthanize the animal now*. (pos. 109)

The lack of understanding evident here was also revealed in physician H1.1's curious question, thus turning the tables: “*But that would interest me as a human physician. Why don't you* [i.e., the veterinarians] *just wait until the animal dies by itself? I mean, you can give painkillers and also sedatives for cramps. Why not just sit it out?”* Like V7.1, he seemed to perceive the standard procedures of his own profession as well-founded and did not come up with obvious reasons for different approaches in veterinary medicine. Some participants noted divergences between the dying of human and animal patients and expressed motivation to diminish these–but only by pulling the other discipline closer to one's own rather than the other way around or by meeting in the middle.

Veterinary oncologist V2.1 confirmed that financial constraints cause manifold problems in (EOL) treatment situations. First, in cases of easily treatable injuries like bone fractures, there are patient owners who are unwilling or unable to pay. For a veterinarian, this equals a dilemma as she is, on the one hand, not able to treat the animal, and, on the other hand, legally not allowed to euthanize it without a justifying reason. Second, in case of rather expensive treatments in oncology that patient owners would like to choose for their companion but cannot afford, those people “*feel as if they are totally failing” (pos. 105)*. Third, she alluded to cases of veterinarians who claim that a medically advanced treatment is “*much too expensive anyway. You don't really do that” (pos. 105)* before even properly explaining the options to patient owners.

V2.1 also–implicitly–confirmed physician 7.1's suspicion that there are, indeed, logistic reasons for not administering palliative care to animals like in human medicine. She observed an increasing number of patient owners who would like to avoid euthanasia and keep their dying animal alive and pain-free as long as possible. The veterinary oncologist elaborated:

“*So what the problem here is that there are a few levels involved: First of all, this person thinks that it is always dignified in human medicine. That may be so; one can leave it that way for the time being.”* Her wording indicated some doubt here concerning human dying processes, but she did not further explain her view in this respect. She continued*: “And that should also be the case of course for pets. But the person does not see that then [in human medicine] a palliative physician visits perhaps constantly, injects high-potency painkillers, that a correct care takes place daily […]. We can't do that at all.”* She continued to describe a gap between medical advances in veterinary and human medicine that clients might not be familiar with: “*They imagine it easier than it is.”* She referred to constraints in “*adequate pain therapy”* or not having the option to “*give morphine to patients to take home,”* concluding that “*if you have already given everything that you can give as an outpatient and there is nothing left, then that is also difficult. And then it is no longer a dignified death.” (pos. 115)*

Finally, she added a reference to biological characteristics to her statement based on her professional experience: “*And an animal, […] doesn't die just like that, it takes forever” (pos. 115)*. Here, again, she pointed toward divergent situations in human and veterinary practice, which cannot be overcome due to substantial differences between the patients, as elaborated in the following chapter.

#### 3.2.2. Differences between human and companion animal patients

Reflecting on procedures in human and veterinary medicine, our participants inevitably looked for differences between their patients–humans and (companion) animals. We identified three emerging themes regarding this aspect:

Theme: Humans and animals are different, for example regarding the ability to express their will or regarding their inner emotional and cognitive lives. Therefore, EOL situations are rather different in human and veterinary medicine.

Some participants suggested that non-human animals perceive their own situation quite differently compared to humans. V6.2 compared her mother “*getting old, she has one illness after another”* to aging animals and concludes: “*You can't put her to sleep or anything. She still wants to live. But I think to myself, an animal doesn't think that far ahead (pos. 78)*. Similarly, H1.2 states she “*would always think: The animal sees it very pragmatically. They have their children, they do it all themselves, they don't think about their puppies or anything else beforehand. The problems are more the owners than the animals themselves […] (pos. 52)*.

Both statements argue for a calm and accepting attitude of animals with regard to their own fate and events in their lives in contrast to humans who sometimes feel or act against what awaits them or others.

Similarly, veterinary nurse 4.2 disagreed that euthanasia situations in human and veterinary practice are comparable, pointing to rather general differences between human beings and animals:

V4.2: “*We simply have the patient who cannot formulate in words what they want and who we also assume is not aware of a future, in the larger sense. So you could try to explain to them what it means: ‘I'm going to put you to sleep now […] or you would have to lie in a basket and be carried out for the rest of your life'. […] It's just not possible for them to assess that. […] They rather live in the moment and that's why it's not comparable for me in any way. The patient's owners are always more concerned about suffering than the animal itself.” (pos. 112)*

Referring to the anthropological difference from the human medical perspective, physician H7.1 stated that in his profession “*we still have the will of the patient. And there is still discrepancy between the relatives' will and the patient's will. That is, I think, another difference here in this case to veterinary medicine, because […] the animal can, of course, not precisely explain his will” (pos. 64)*.

Strikingly, despite the intention to point out differences, both statements referred to third parties, animal owners and patients' relatives, who judge the situation differently than the animal or human patient, respectively.

Theme: Humans and animals are not that different; experienced veterinarians can identify their patients' preferences from, for example, the animals' behavior in a way that makes EOL situations quite comparable to those in human medicine.

Having insight into animals' preferences is not unachievable according to experienced veterinary oncologist V2.1:

“*[…] that may sound bizarre, but there are animals that you can tell, especially if you've been taking care of them for a long time, when they can't take it anymore. They have a very odd look in their eyes. […] especially those that you've known for a long time and that you've looked after […] and that always happily run in and out and have always shown a certain behavior / And then it's no longer like that and it's not easy to fix; then it's easier to say ‘He doesn't really want to live anymore'. That's how we express it under certain circumstances, even though he hasn't said that he doesn't want any more.” (pos. 95)*

Theme: Certain animal and human patients share relevant characteristics such as not being able to explicitly communicate their preferences, having only an idea of the very immediate future, and not being autonomous. Treating those patients is quite comparable in human and veterinary medicine.

One of the most important guiding factors in human medical decision-making is the human will or ability to express preferences. The case example allowed our participants to consider both general differences between most human and most animal patients and commonalities between particular (groups of) human and animal patients.

Nurse H4.1 picked up that cue in a concise statement: “*The problem in intensive care is that not all patients are able to make their own decisions. And that, of course, is a parallel to the animal sector for me, because, of course, it's not the patient who decides. The animal […] does not make the decision. […] it is on behalf of the actual patient that decisions are made and spoken” (pos. 86)*.

Veterinary nurse V3.1 added: “*It is somehow a strong parallel to veterinary medicine, I think, because there the patient has no say at all, and they cannot decide anything. Others have to decide for them and yes, an agreement with the patient is not possible and they can also not express their will.” (pos. 131)*

These statements reveal that not only the patients' characteristics but also the constraints concerning interaction between caretaker and patient and the affliction of relatives can be similar.

#### 3.2.3. (Critical) reflections on both professions in the light of the interdisciplinary exchange

When asked about their impressions after the discussion, most participants from the veterinary profession declared to have been fairly familiar with the human medical perspective beforehand, mostly due to personal experience, so that examples and procedures were not entirely new to them. Nevertheless, they evaluated the discussion as having been fruitful and interesting. Participants from the human medical field, in contrast, more frequently reported to have learned something new about the other discipline.

Theme: Unexpected parallels

Several participants were surprised to realize that there were so many parallels between human and veterinary medicine, having expected more fundamental differences. Starting with basic insights like “*actually everyone wants a good death for humans and animals” (H5.2, pos. 228)*, the discussants referred to different parts of the conversation when reflecting on their attitude toward their own profession, and, in particular, to the examples provided by the interview guide or by other participants. They mentioned unexpected similarities regarding the “*people [who] are involved in the decision to end a life” (V4.2, pos. 201)* or the “*attention [that] is paid to such little things […] to whether there was a cuddly toy” (V5.3, pos. 229)*, concluding “*especially when you see your relative for the very last time or your pet […] the tendency is that we all understand that and that we make it as pleasant as possible. Especially for the bereaved, simply. It doesn't matter whether they are patient owners or relatives” (ibid.)*.

Occasionally, both parties claimed to take home some inspiration or input from the other discipline. Several times, participants stated that the discussion was thought-provoking and gave reason to further conversations, reading, or thinking about EOL situations in interdisciplinary ways (“*you should draw more comparisons there,” V4.1, pos. 198; “one could also work a bit more in both areas on the way dying is dealt with,” H5.2, pos. 228)*.

Theme: Euthanasia as active killing affecting veterinarians

Strikingly, veterinarian V7.1, who dedicatedly discussed differences in the attitude of physicians and veterinarians when it comes to killing patients, concluded with a far more contemplative statement as if having thought about or even doubting her own perspective in the course of the conversation:

V7.1: *Well, what I would perhaps also be interested in is of course the view of a veterinarian, to what extent a veterinarian is allowed or should euthanize animals at all. This is also an ethical question that veterinarians have increasingly been dealing with in recent years. How does it affect a veterinarian that he simply takes the right to euthanize animals*. (pos. 185)

Family physician H1.2 quoted a more drastic description of the veterinarian “*as an executioner*” when euthanizing patients, emphasizing the contrast between active killing in veterinary medicine and allowing and accepting death in human medicine:

H1.2: *You really do something. That is, I think, more stressful than with humans; that you go there and really do it*.

V1.2: *It is an active act of killing. I don't take a knife and stab it, but I take a needle, stick it into the animal and then the animal stops breathing*.

H1.2: *Yes, the other thing is more unburdening; I really have to say that. (pos. 246-248)*

Similarly, veterinary nurse V4.2 identified the burdensome situation for veterinarians:

V4.2: *So, the decision to let someone go, whether by euthanasia or by stopping the therapy, is, I think, something that is never easy, that is often simply attributed to the veterinarians, you just put them to sleep. What should not be underestimated is the burden that ultimately lies on those who feel they have to make this decision […]. (pos. 207)*

The field of tension between the relief several veterinary discussants mentioned due to the option of euthanasia and the burden they may feel when having to decide and act became evident in the final reflective part of discussion of the focus groups.

Theme: Professional relationship of veterinary and human medicine

Identifying additional convergence, V1.2 felt that veterinary medicine is gradually adopting concepts from human medicine, for example, when it comes to “*saying goodbye. I think this is something that will perhaps also become more important for us in veterinary medicine. Simply because this relationship between humans and pets is changing and the animal is becoming more and more important. And also in the direction of hospice and so on, there are more and more voices saying ‘No, my dog should die as naturally as possible”' (pos. 250)*.

A few participants admitted having had prejudices against the other discipline: H6.1 expected veterinarians to “*over-romanticize”* and was surprised to learn “*how factual [they] argue about it” (pos. 210)*. Veterinarian V 2.1 had “*always thought, ‘Thank God I became a veterinarian. I can at least take suffering”'* but learned in this group discussion about human medicine “*that it's not quite so trivial and that it's not always just: ‘We'll do everything and every chemo, no matter what”' (pos. 212)*.

## 4. Discussion

### 4.1. What was gained from the choice of methods and mix of materials?

Two examples will be singled out in the following. The “cross-questioning” of the participants (TV clip and newspaper article) excelled expectations, whereas letting participants comment on fictitious (provocative) statements did not work as expected but still yielded interesting results (see 4.2).

Cross-questioning participants worked very well for the TV clip: Human medical professionals appreciated the bluntness of the vet, (partially) emphasizing the advantages of such a bold communicative style compared to their own profession. Most human medical professionals accepted the presented situation as being suitable for veterinarian practice and only mildly criticized the TV vet's tone or wording. It was “OK for veterinary medicine.”

The participants with a background in veterinary medicine, in contrast, did not appraise this as a typical, acceptable or a good communication example for their profession. In contrast to the participants from human medicine, they saw their own “gold standard” as being much closer to human medical practice (involving patient owners in decision-making, presenting all options, choosing gentle wording, an appropriate setting etc.).

There are several conceivable explanations as to why the participants with a background in human healthcare deviated from the (veterinary) professionals' opinion. Most plausible from our point of view is a mixture of reasons: Several human healthcare providers disclosed unfamiliarity with small veterinary practice.

Our course of action deliberately put participants on the spot by asking them for their impressions right at the beginning of the discussion when participants were unacquainted with each other and in the presence of veterinary professionals. They later expressed surprise regarding how similar EOL care in both professions is, for example. It is conceivable that this course of action led to more “cautious” (i.e., less critical) responses on behalf of those with a background in human medicine.

This is in line with previous findings suggesting that the gap between human and veterinary medicine is perceived as wider by human medical professionals than by veterinarians, for e.g., (24, 25). Veterinary medicine usually learns from human medicine and not vice versa and human medical providers are thus much less familiar with medical, communicative and ethical procedures as well as standards in veterinary medicine than contrariwise. The fact that this asymmetry cannot be found in the second example introducing a case from human medicine (see 3.1.2 for details) supports this interpretation.

At the same time, professionals from human medicine express the wish to share veterinarians' “freedom” regarding clear communication and steering the decision-making process in a way that leads to medically indicated treatments (or termination of treatments). This goes in hand with the hypothesis of converging ideals of death in both professions (15).

The results regarding the newspaper article revealed different patterns and showed fracture lines between sub-groups (physicians/veterinarians vs. nurses/veterinary nurses).

The procedure of the Dutch doctor from the newspaper article was harshly criticized by physicians and veterinarians, whereas the (veterinary) nurses were much more ambivalent in their assessments. The participating physicians and veterinarians had on average much more working experience than the (veterinary) nurses, possibly positively affecting confidence in personal and professional (ethical) judgments (26). Interestingly, this professional self-assuredness also included the veterinarians for whom the case represented a scenario from the respective other discipline. Their criticism, however, concerned less human euthanasia in general but rather mainly focused on the technical aspects and other specific details of the case. Human euthanasia, if accepted, should follow certain criteria (suffering, patient's will); convenience euthanasia should not be tolerated. Thereby, criteria that are decisive for animal euthanasia were transferred to human medicine without making this transfer explicit.

Commenting on a statement of a (fictitious) person outside the group gave the focus group the opportunity to collectively distance itself from a certain position, because participants automatically needed to reflect their own beliefs and assumptions. However, it turned out to be unsuitable for bringing participants out of their shells and provoking open disagreement in the conversations themselves.

### 4.2. Animal hospice and palliative care

Animal hospice (27) (as a practice, not a location) is a comparatively new field in (German) companion animal medicine (28), mirroring the overall tendency to transfer treatments, approaches, and attitudes from human to veterinary medicine (29, 30). Measurements cover advanced pain management and palliative care, counseling regarding aids and tools for geriatric, handicapped or terminally ill patients, but also deep sedation as an alternative to euthanasia. From the perspective of animal hospice, a palliated natural death, if requested by the animal patient's owner, is acceptable as long as the animal is comfortable and not in pain (23). There have been only a few attempts made at approaching veterinarians' perspectives concerning animal hospice and care perceived as medically futile or non-beneficial, for example (31, 32). Small animal practitioners in the US commonly encounter requests for what veterinarians deem as futile care but also agree that there are situations where its provision is appropriate (31). However, veterinarians disagree on whether palliative sedation is appropriate EOL care (32). Confronting participants with an anti-euthanasia statement was meant to sharpen the arguments on both sides: Why do we choose or recommend a treatment for (a certain group of) patients? Why do we reject particular options?

Expected arguments regarding terminal care as an explicit alternative to euthanasia raised in the discussion could roughly have followed one of the patterns stated below:

- Hospice-supported natural death is for humans and not for animals

This first argumentative pattern was not detected in the participants' reactions to the fictitious patient owner's statements. Given that it reflects the current standard in human and veterinary medicine, this might be surprising at first glance. At second glance, though, the statement was introduced at a relatively advanced point in the discussion. Participants had already exchanged their views on human euthanasia after the newspaper article and might be less inclined to fall back on, for example, the otherwise common argument of human exceptionalism to argue for their position. Furthermore, the argument might not be perceived as appropriate in the context of the interdisciplinary group. Much like in the first reactions to the TV vet (see 3.1.1), human medical personnel is potentially not aware of customs, practices and most common attitudes in veterinary medicine, and wants to avoid making a faux pas, due to social desirability bias. An implicit hint of an important difference in human and animal dying and, therefore, in EOL decisions was given by the vet noting that the dying of cats, if not euthanized, “*take[s] forever.”* Human medical professionals did not pick up her argument, though, which is why an exchange on the length of dying processes did not take place either.

- Hospice-supported natural death is not even good for humans (in some cases); likewise, therefore, not for animals

The second argumentative pattern is based on practical experience rather than on theoretical assumptions regarding humans and animals. Negative incidents with human deaths in hospitals led two participants working in ICUs to follow this argumentation. Shortage of nursing staff and, consequently, limited time for patient care present two well-discussed challenges in hospital patient care. More specifically, though, medical research progress and the increasing proportion of highly aged patients culminate in a growing group of patients in ICUs that suffer from prolonged dying processes (33), also referred to as “dysthanasia” (34, 35) and what is termed “chronic critical illness” (36). Being aware of the overall tendency of veterinary medicine to follow in the footsteps of human medicine, these two participants seem to warn veterinarians to critically scrutinize procedures and practices before adopting them in their own repertoire. This perspective is a valuable addition to the dominant image of a “good death” under palliative care. After all, the treatment of dying patients in companion animal clinics might share limitations and constraints with procedures in human ICUs, as suggested by recent research on dysthanasia in veterinary practice (37). Those very specific questions were, however, not dealt with in our study.

- Euthanasia is better for animals than hospice-supported natural death

This pattern focusses on assumptions regarding animals and properties thereof that make a quick death preferable over mere medical support during the final phase of life. V2.2 described experiencing an increase in demand for terminal palliative care as a replacement for euthanasia and harshly criticized this trend. While we did not learn about her reasons here, ICU nurse H5.2 explained her doubts about the treatment options for animals: We are not able to access the animal patient's preferences in this regard. Concluding, accordingly, that in some cases, the “*faster variant would be more humane”* she pointed to what might be called the *euthanasia paradox*: In human medicine, in cases of uncertainty about the individual preferences of a patient, they are kept alive. In veterinary practice, in contrast, in case of doubt about the animal's preferences, their life is terminated. The fact that we do not know the patient's preferences thus leads to quite different decisions being made. It would be simplistic to claim that the legal difference between the protection of a human and the protection of an animal life was the main reason. The nurse's statement rather suggests that keeping the animal alive under palliative care might cause more harm than benefit for the patient, which is closely linked to euthanasia justification patterns for veterinarians (14). In veterinary practice, euthanasia is considered one of many treatment options that is performed–like other treatments–if indicated; even if, according to Quain, “[w]hat constitutes ethically indicated veterinary euthanasia is by no means clear-cut” (38). With it being (a quite powerful) part of their toolbox, veterinarians might prefer its unquestionable success in the relief of pain and suffering to the uncertainty of pain relief with the help of palliative measures and unclear side effects for the animal's wellbeing.

- Hospice-supported natural death is a good option for animals.

The alternative animal health practitioner V7.2 and the geriatric vet V7.1 considered the fourth argument, as they had a lot of experience in accompanying patients and their owners in the animal's final phase of life. V7.1, however, revealed a critical attitude toward palliative sedation for companion animals at a different point in the discussion (see 3.2.2). The woman's statement on the slide was intentionally left imprecise. How exactly she wanted her dog to be treated in his terminal phase is unclear, which is why our discussants collaboratively saw the lack of established alternatives to companion animal euthanasia as one major obstacle. Like in human medicine with several treatment options in palliative and hospice settings, the question *how* terminal care can be provided in veterinary medicine seems to be more fruitful than *if* it can be provided.

### 4.3. Good death and dying for humans and companion animals

That (veterinary) nurses in our study much more frequently merged the perspectives of all involved parties when deliberating about aspects of a good death than veterinarians and doctors, is, at first glance, incongruous with recent findings that nurses are primarily concerned with the patient's perspective, while physicians place much more emphasis on, for example, the family's needs (39, 40). On closer inspection, however, our results can be seen as complementary. Both nurses and physicians seemingly viewed themselves as advocates of their patients, but interpreted their role differently: Nurses regarded themselves as spokesperson for their patients (39) *(p. 2025)* and (40) *(p. 638)*, occasionally fighting for the acknowledgment of their needs. They strongly identified with their suffering (40) *(p. 637)* and even “*feel that they alone understand patients' feelings because family members are overwhelmed by emotions and are thus unable to evaluate the situation”* (21, 39) *(p. 2025)*. They also identified with the family's emotions and experiences and “*consider themselves to be close relatives” (ibid.)*. They wanted to treat the patient as they would want to be treated. Their reflections on the good death from the patient‘s perspective will therefore inevitably be mixed with their own ideas about the good death from a personal and professional perspective as well as first and third person perspectives. Nurses, therefore, may (un)intentionally merge the perspectives and emotions of all involved parties when thinking about good dying.

Physicians, in contrast, may explicitly try to avoid a merging of perspectives in EOL decision-making. This is compatible with findings that physicians place greater emphasis than nurses on the needs and wishes of the family than nurses do, because they consider the family their primary source of information about the patient's wishes but also as a rational object of moral concern.

The two above-mentioned findings–difficulties with the task and the merging of perspectives–make it difficult to interpret several other results. Among them the outcome that participants arranged many key words during the Padlet task in the middle, indicating their applicability to both human and animal patients. When deciding against a “*death at home”* as component of a good animal death because of the traumatic impact this had on the animal's owner, it remains unclear whether the respective group:

- Actually intended to think about how (and where, in this case) an animal would prefer to die but was unable to disentangle the perspectives of all involved parties; or
- Followed a unit of care approach (41), deliberately though maybe subconsciously merging the perspectives of all those involved in the dying process. For instance, V3.1's assessment to put all key words in the middle, including, for example, “*self-determined”* and “*spiritually accompanied,”* may plausibly be interpreted as an indicator of a unit of care approach.

In a similar fashion, what allows for several interpretations is our finding that there was no consensus regarding the question whether acceptance of death is part of an ideal good human death. In light of some groups' uncertainty regarding the task and the fact that there was cross-group consensus that good dying is, above all, a matter of individuality, we consider the following argumentative patterns as most probable:

- Acceptance of death is necessarily part of a good human death;
- Acceptance of death is not a necessary part of a good death because death ideally is sudden and unexpected;
- Some deaths are so tragic that they cannot be made “good,” regardless the dying person's efforts.

The first argumentative pattern was most clearly brought up by three nurses, H5.1, H5.2, and H4.1 (see 3.1.4). The acceptance of death, just like the denial of death, are understood as necessary phases in the course of dying, by which they seem to refer to the phases of grief by Kübler-Ross (42). This is in line with the palliative good death ideal, having its roots in the beginnings of the hospice movement in London in the 1960's. Denials and irrational attempts to defeat death are being rejected; efforts to accept death as a natural part of life promoted (15). Once again, the perspectives of the patient, their family, and medical team merge in the participants' remarks, making it partly unclear for whom an acceptance of death is beneficial. This connects to recent criticism, arguing that the here depicted good death ideal is too rigid, cannot account for individual preferences and puts too much pressure on the dying patients to commit to the “right” way of dying (43).

In contrast, V6.2 argued along the second argumentative pattern, stating that acceptance is not part of good dying because dying ideally occurs suddenly and unexpectedly. This is in direct opposition to H4.1, who was convinced that acceptance of death as part of dealing with one's own mortality is an important part of being human and, possibly, also dying like a human. In emphasizing that for her as a daughter, the unexpected death of her father was tough but she also found peace in the thought that this death was really good for her father, V6.2 made it explicit that for her, considerations of a good death are a matter of the patient's perspective alone. This was also shown throughout the interview in her assessment that it is first and foremost the animal's perspective that matters in EOL decisions and situations and not the owner's or the veterinarian's.

Finally, and in line with the third argumentative pattern, oncologist H2.1 explained her belief that some deaths are just so terrible that they do not lend themselves to pursuing a good ideal of death. She also indicated that a denial of death is incongruous with a good death, thus expressing two separate concepts of non-acceptance: One, as being unable to find peace with one's own fate through non-acceptance, and two, in terms of denying one's situation and fate.

In order to narrow down the discussion of differences and similarities in human and animal patients, we want to focus on what is known as the argument from marginal cases (44–48) or the argument from species overlap (49). Generally, the argument works as follows: While there are differences between most humans and most animals, there are cases of certain humans–the “marginal” cases–who share their morally relevant properties with certain non-human animals. This group consist, for example, of infants and persons with dementia or other mental handicaps. Like other animals, they cannot act autonomously, they are not able to tell us about their preferences and we are not able to explain future benefits from current treatments or actions to them. At the same time, both animal patients and the so-called marginal cases are able to suffer and clearly express suffering.

By providing an example of a demented woman who is, in the end, euthanized, the discussion could have been navigated toward the commonalities between ending a patient's life in human and veterinary medicine, i.e., toward the marginal cases debate. Alternatively, the scenario could have induced a discussion about the differences or divergences between the two fields. The presented results suggest that both variants occurred: Some participants agree that the situation is similar in veterinary practice. They stress the observable behavior in the actual situation: Animals–like the woman–are fighting back while those who are competent to judge the situation (doctors and family) decide that it is allowed or required to end their lives. This is mirrored by the fact that in the current debate on respecting autonomy in veterinary practice, the focus is rather on the patient's owner's than the animal's autonomy (50). Other participants state they would not euthanize animals who are still able to fight back that way. Still, they judge the situation as comparable. One should not have killed the woman as we would not have killed an animal in that situation.

In contrast, the position of the (veterinary) nurses implicitly draws a line between the once autonomous woman who is killed because she previously expressed her will, and animals, who are killed for different reasons. This line of argument points toward crucial specifics of the woman's case that are incompatible with decision-making in veterinary practice. Having an advance directive shifts the justification of the professional decision from the principle of beneficence to the principle of autonomy, if applying the standard principles of biomedical ethics (51). Several participants uttered they could not detect any signs of suffering in the patient–which would have been the main criterion in veterinary practice. The decision was justifiable, if assessed as respect for autonomy, according to the patient's formerly expressed will. The ambivalence in this case is reflected by the considerable disagreement in the literature on whether in this and similar scenarios the previously communicated autonomous will should be decisive in decision-making (as is currently the case in the Netherlands) or the (verbally and non-verbally expressed) preferences of the current but no longer autonomous patient should precede [see (52) for an overview of arguments and positions].

The fact that there was no agreement on the (anticipated) question whether the case is comparable to animal euthanasia might as well be due to some general blurriness regarding the justification for animal euthanasia in veterinary practice [see (14)] as indicated by V7.1's pondering statement at the end of the discussion.

Consequently, the severely demented patient from our case and a hypothetical animal patient can be perceived as more or less similar, depending on the chosen criteria that (do/do not) justify their euthanasia. Altogether, the discussion initiated by the example of the demented woman turned out to be fruitful against the background of the professional heterogeneity. A clearer focus on the argument from marginal cases could have been achieved using a thought experiment rather than a real-life scenario. After all, the background story and personal context were not easily transferable to a patient in veterinary practice.

### 4.4. Critical reflection on both professions and respective EOL processes

In our focus group discussions, interdisciplinary considerations do not only occur when a participant comments on an incident or perspective from the other profession. Rather, they are also an expression of the intertwinement of professional and personal experience when it comes to questions of death and dying. A veterinary nurse can additionally be the grandson of a woman with dementia. An oncologist might have accompanied her family dog in his final hours. Not least, all participants might have thought deeply about their own mortality and their preferences in this regard. Judgements on a good death and good dying are therefore not necessarily merely professional.

Whereas, being confronted with their own (i.e., human) mortality is a natural challenge to all our participants, human medical personnel have not necessarily dealt with dying companion animals before. The resulting imbalance concerning familiarity with crucial issues of the other field presents a potential explanation for veterinarians talking more freely and judging more easily than human medical personnel about the respective other discipline.

On the other hand, familiarity with the respective other discipline also poses a risk for potential misunderstandings to arise or certain prejudices to be confirmed. Professionals from veterinary medicine were occasionally oblivious to the specifics of human medical ethics and remained convinced, for example, that the focus in human medicine is on prolonging life rather than the quality of life.

The exhaustive exchange between V7.1 and H7.1 clearly illustrates that comparing procedures, attitudes, and legal constraints in both disciplines is challenging and by no means straightforward. It also reveals an attitude that might not be uncommon among professionals of both disciplines although no other group discussed this aspect explicitly (see 4.1.3). Albeit they are medical professionals who have in common a long and thorough education, a good reputation, a great deal of responsibility for others' lives and the wellbeing of patients and their families, there are also fundamental differences between the veterinarians' and human physicians' attitude toward and understanding of their profession. This becomes evident, for example, when weighing the value of life against the value of quality of life in EOL decision-making, but also in the way quality of life is and can be assessed (6, 53, 54). An international exchange between professionals from countries with different legislations regarding animal and human euthanasia would be promising.

Given our experience in the recruitment process, we assume that there is a group among veterinarians and veterinary students who are trained nurses but who changed the professional field. For future interdisciplinary research, this group could be of particular interest regarding their experience in two different healthcare sectors and their motivation to change.

This study has several limitations. Limitations linked to the mix of methods and material applied in the interviews, and to the heterogeneity of the groups have been addressed above. Other limitations included difficulties regarding the discussion inputs: Several participants questioned the authenticity of the TV-vet and expressed doubts as to which scenes were “real” and which were scripted. Regarding the second case example, some participants showed difficulties in ethically evaluating a case from a country (Netherlands) with a different legal framework as they were not familiar with any cases of killing on request. Due to the restrictions during the pandemic, the focus groups (with the exception of the first one) were online, resulting in a relatively young field of participants, especially regarding (veterinary) nurses. Other limitations refer to an imbalance between the two professions due to an inability to recruit as many physicians as veterinarians, and a limited generalizability of our findings due to, for example, the specifics of German national law. Finally, in future research, a further elaborated methodology like the Reflexive Thematic Analysis by Braun and Clarke (21, 55) could expand and refine results from qualitative data.

## 5. Conclusions

In accordance with previous work by the authors, the data obtained in this study represent further evidence that discourses and ideas related to EOL situations in veterinary and human medicine converge–often without their representatives being aware of it. Concepts, treatments and ethical principles are being transferred from human to veterinary medicine, human medicine is confronted with requests for physician-assisted suicide and generally more diverse death ideas. Our analysis reveals that treating dying companion animals is accompanied by attitudes, challenges, and obstacles that largely mirror EOL situations in human medicine–often to the surprise of the involved parties. It also became apparent that there are occasional misconceptions of EOL care in the respective other profession.

The mutual benefit through the exchange was pointed out by participants from both professions in the herein presented focus groups, relating above all to the elimination and correction of prejudices and concepts. As exchange between professions on matters of EOL care and ethics is still rare, concrete benefits for the work of practitioners are only at the horizon. This being said, we expect that collaborative work on old and emerging EOL concepts, in constant reflection of the similarities but also the differences between (human and animal) patients such as (animal) hospice, palliative sedation or euthanasia can avoid serious misconceptions and hence will also affect practitioners and their patients in the long run.

We conclude that further exchange between the two professions would be valuable for professionals and patients in both fields and could stimulate previously overlooked insights within the academic discourse. Therefore, projects fostering interdisciplinary investigation, cooperation and communication should be further promoted, especially with regard to ethical challenges and possible solutions. Future research should examine, for example, if learning from the interactions between EOL discourses in both professions can contribute to the reduction of moral stress for both, veterinarian and human medical staff.

Overall, the conception and materials applied in this study proved to be suitable for sparking thesis-driven, interdisciplinary discussion. The study design can be refined as a basis for further research in this important area.

## Data availability statement

The raw data supporting the conclusions of this article will be made available by the authors, without undue reservation.

## Ethics statement

The studies involving human participants were reviewed and approved by the Animal Welfare Officer, Member of the Research Ethics Committee of the University of Veterinary Medicine Hannover. The patients/participants provided their written informed consent to participate in this study.

## Author contributions

KP, FS, PK, and GN: conceptualization, methodology, and writing—review and editing. FS and KP: formal analysis, data curation, and writing—original draft preparation. KP: visualization. GN and PK: supervision, project administration, and funding acquisition. All authors have read and agreed to the published version of the manuscript.
