# Supplementary material for: End-of-life decisions: A focus group study with German health professionals from human and veterinary medicine
Source: Front Vet Sci. 2023 Feb 15;10:1044561. doi: 10.3389/fvets.2023.1044561 (PMC9975587; doi:10.3389/fvets.2023.1044561)
Supplement: Supplementary file 1 [file Data_Sheet_1.PDF]

Data protection information and declaration of consent for the focus group study of the DFG project "Dying like a dog". (Translated from German with www.DeepL.com/Translator (free version))

1. Purpose of the data processing
We collect and process the personal data for participation in the focus group study mentioned above. The purpose of this study is to analyse convergences and divergences in end-of-life situations in human and veterinary practice. In line with current guidance on avoiding face-to-face contact, the study will be conducted online using the Microsoft Teams collaboration platform. Video recordings will be made of the focus group discussions conducted. The platform is password-protected and the discussion channel can only be accessed by the project staff or study participants. The backup stored in the Microsoft Cloud is also only accessible to the project leaders and study participants. The video footage will not be used for the analysis. The audio track of the recording will be anonymously transcribed afterwards. The transcribed, anonymised data will be analysed using the methods and tools of qualitative social research. The results of the study will be used by those responsible for the study for research, presentation and publication purposes; if personal information is available, it will be anonymised beforehand. Art. 6 I 1 lit. e i.V.m. 89 DSGVO, § 3 I 1 Nr. 1 NHG, § 13 NDSG.

2. Legal basis of the processing
Participation in the focus group study is voluntary. If you are employed at the institutions conducting the study, you will not suffer any disadvantage with regard to your employment relationship as a result of participation or non-participation; rather, the study does not affect your employment relationship as such. Likewise, it can be assumed that the responsible body and the employees pursue the same interest, Art. 6 I 1 lit. a, 7 DSGVO in conjunction with. § 26 BDSG i.V.m. § 12 NDSG. In particular, the focus group discussions are video-recorded, so that personal data are processed at this point. The processing can only take place through your voluntary and informed consent, which we obtain at the end of this declaration, Art. 6 I 1 lit. a, 7 DSGVO. You can revoke your consent at any time with effect for the future, Art. 7 III DSGVO.

Microsoft Teams provides its own data protection declarations, which can be accessed via the following link: https://www.microsoft.com/de-de/microsoft-365/microsoft-teams/security.
Pursuant to Article 28 of the GDPR, Microsoft processes personal data via Microsoft Teams on behalf of the University of Veterinary Medicine Hannover Foundation. There is no contract within the meaning of Art. 28 III DSGVO; however, Microsoft provides with its Online Service Terms (OST) as well as the Online Services Data Protection Addendum (DPA) an "other legal instrument [that] binds the processor in relation to the controller and sets out the subject matter and duration of the processing, the nature and purpose of the processing, the type of personal data, the categories of data subjects and the obligations and rights of the controller". The OST can be found at https://www.microsoftvolumelicensing.com/Downloader.aspx?documenttype=OST&lang=German and the DPA at
https://www.microsoftvolumelicensing.com/Downloader.aspx?DocumentId=16042
as amended from time to time.

3. Duration of the processing
The personal data will be stored for the duration of the study and the research project beyond. Deletion takes place automatically, at the latest 6 months after the end of the project. Legal storage obligations remain unaffected. In the Microsoft Cloud, the stored video file is deleted immediately after downloading and creating the transcript.

4. Rights of the affected
You have a right of access to the personal data concerning you and, if applicable, to rectification,

erasure or restriction of the processing of this data and a right to object to the processing as well as a possible right to data portability vis-à-vis the institution mentioned below. You can revoke your consent, if any, at any time with effect for the future, Art. 7 III DSGVO In addition, you have the right to lodge a complaint with the supervisory authority if you are of the opinion that the processing of personal data concerning you violates the legal provisions. The competent supervisory authority is the State Commissioner for Data Protection of Lower Saxony.

Head of the institute<br><br>Prof. Dr. Nicole Kemper<br>Institut für Tierhygiene, Tierschutz und Nutztierethologie<br>Bischofsholer Damm 15 (Gebäude 116)<br>30173 Hannover<br>Tel.: +49 511 856-8951<br>E-Mail: Nicole.Kemper@tiho-hannover.de	Project leader<br><br>Prof. Dr. Peter Kunzmann<br>AG Angewandte Ethik in der Tiermedizin<br>Stiftung Tierärztliche Hochschule Hannover<br>Institut für Tierhygiene, Tierschutz und Nutztierethologie<br>Bischofsholer Damm 15 (Gebäude 116)<br>30173 Hannover<br>Tel.: +49 511 856-8956<br>E-Mail: Peter.Kunzmann@tiho-hannover.de
Contact person<br><br>Dr. Kirsten Persson<br>AG Angewandte Ethik in der Tiermedizin<br>Stiftung Tierärztliche Hochschule Hannover<br>Institut für Tierhygiene, Tierschutz und Nutztierethologie<br>Bischofsholer Damm 15 (Gebäude 116)<br>30173 Hannover<br>E-Mail: Kirsten.Persson@tiho-hannover.de	Data Protection Officer University of Veterinary Medicine Foundation<br><br>Wolfgang Rottwinkel<br>Bünteweg 2, 30559 Hannover<br>Tel.: 0511 953 8015<br>Fax: 0511 953 828015<br>E-Mail: datenschutz@tiho-hannover.de

Informed Consent

I,

____
(Name in block letters)

hereby consent to the above-mentioned video recordings being made of me, used for the aforementioned purpose and used as listed. I do not derive any rights (e.g. remuneration) from this consent to use. This declaration of consent can be revoked at any time with effect for the future, Art. 7 III DSGVO. In the event of revocation, the recordings will be removed from the respective platform. If the recordings were available on the internet, they will be removed insofar as this is subject to the initiator's possibilities of disposal. I have received the enclosed information sheet with the data protection information on consent.

Place, date: ______________________ Signature: ______________________
